# Selections and Abstracts

**Published:** 1915-04

**Authors:** 


### SELECTIONS AND ABSTRACTS

### TUBERCULOSIS "REMEDIES" THAT ARE WORTHLESS.

### No Real Cure Possible From Any of the Patent Preparations Investigated by Government Scientist.

Washington, D. C.—After investigating under the Food and Drugs Act, a large number of preparations advertised as consumption cures, the Department of Agriculture has not been able to discover any that can in any sense be regarded as "cures" for tuberculosis. Some contain drugs that may at times afford some temporary relief from the distressing symptoms of the disease, but this is all. Since the passage of federal legislation prohibiting the shipment in interstate commerce of medicinal preparations for which false and fraudulent claims are made, there has been a marked tendency to label these preparations "remedies" instead of "cures" or "infallible cures," as they used to be called. In many cases, however, they can not even be regarded as remedies.

A "cherry balsam," for example, for the "cure" of "consumption" and "hemorrhage of the lungs," which it was represented would "strike at the very root of the disease" was found on analysis to be nothing but a solution in water and alcohol of opium, sugar, benzaldehyde, inorganic salts and coloring matter. It contained no cherry bark extract or balsam.

A more elaborate "cure" consisted of five different preparations which the credulous patient was to take separately. These were first, the medicine proper, the essential ingredients of which were found to be morphine, cinnamic acid and arsenic —not a very safe mixture to take habitually; second, a tonic which was supposed to contain iron, but did not; third, a "cough mixture" made up of alcohol, chloroform and codeine which is a derivative of opium or morphine; fourth, a mixture which contained some quinine, and a solution of water and alcohol; and fifth, codeine tablets. Even the strongest constitution could hardly stand a prolonged course of such a treatment.

In the marketing of such preparations considerable ingenuity is frequently shown. One of the main objects is to persuade the patient that he is receiving, at a comparatively low price, the individual attention of a trained specialist. For this purpose, symptom blanks are employed. These contain a number of questions about the patient's symptoms, the number varying from a dozen or so to as many as 70 or 80. The patient is led to believe that the information which he furnishes in reply to these questions, will be carefully considered before any medicine is prescribed for him, though every physician knows that an accurate diagnosis cannot possibly be made in this way. As a matter of fact none is attempted and the degree of attention which these individual reports receive can be measured by the fact that cases have come under the observations of the department in which mail order concerns doing a business of this kind have received as many as 4,000 letters a day.

After the patient has submitted his "diagnosis report," he is urged to purchase a supply of the medicine. If he does so, he is then urged to purchase more. If he states that he has experienced no beneficial effects he is told that he has not taken enough, and this process is likely to continue until the limits of his credulity have been reached. If, on the other hand, he decides at the beginning not to purchase the medicine it is likely to be offered to him at successively lower prices until he is at last induced to believe that he cannot afford to ignore such a bargain. This is carried to such an extent that a "treatment," the original price of which is $25, may be offered at the end of six months for $2.50.

As a matter of fact the successful treatment of tuberculosis requires much more than the mere giving of medicine, and, moreover, what will help one case will not necessarily help another. Claims that are absolutely unwarranted are no longer permitted on the labels of medicines shipped in interstate commerce, but the wording may be such as to convey a misleading impression without the use of absolute statements. Thus these preparations continue to find a sale despite the fact that a little trouble on the part of the prospective purchaser will reveal their worthlessness.

---

### ULCER AND CANCER OF THE STOMACH. THE CAMPAIGN AGAINST CANCER.

### Symposium: Looking to the Increased Knowledge Of Cancer.

### THE PRESENT STATUS OF CANCER RESEARCH.

By Leo Loeb, M. D., Philadelphia.

(Continued from Last Number.)

Usually, however, the patients of the last two groups have tumors and other symptoms sufficient at the time of presentation to rather easily meet the requirements of diagnosis, but they are generally poor subjects for comforting surgical statistics. Exploration, inoperable carcinoma with wide gland infection are too often the only annotations at operation.

Ulcer is the great soil upon which cancer is engrafted. When ulcer is latent, cancer may develop and no sign be given until such wide destruction and infection are present that surgery is worse than useless. The general picture presented by the patient is often valuable. Wasting and weakness are complaints, and lack of appetite fails to account for all. The picture of cachexia is present, and bears little relation to the wasted appearance of ulcer starvation. Depression seems to be an early sign. The facial expression is significant with pallor about the eyes, nose and mouth and a drawn pinched look. There is a silent fear, with apprehension of grave trouble. This, with depression of spirits, tardy confession of symptoms, slow and weakened movements and listlessness will often leave little doubt as to the presence of cancer.

Pain, a common symptom, is perhaps not so often noted as in ulcer. It is epigastric, rarely acute except when perforative, a dull sickening, indescribable pain; or a strange distress supplants the decided pain-spells of ulcer. It is not as closely related to food, though modified by it. It is a continuous pain or distress, immediately intensified by foods or drinks, though in a few instances, if no obstruction be present, a short relief does follow food if the hydrochloric acid continues high or abstinence has been practiced for a time. The acute pain of ulcer is gone, the time lengthens almost to continousness, and as a rule it is a dull, sickening, distressing, depressing nervous headache. Pain, unlike that in ulcer, is not controlled by foods, but careful diet, bland liquids or abstinence reduces discomfort to a minimum. Subjective localization of pain is not dependable in ulcer, and is less so in cancer; epigastric distress only is the usual diagnostic help in either ulcer or cancer. Vomiting gives relief to the distressing symptoms in cancer as does lavage when carefully practiced.

A good appetite may remain late in some instances, but a decrease of it is usual early, and later, poor appetite is a rule. Fear of food comes correspondingly earlier than in ulcer, and is often a factor of lessened food intake rather early in the degenerating process. A small amount of food seems to fill the patient and gives a bloated, disagreeable oppression. An utter distaste for food is occasionally present, even when early symptoms are noted, and later it is not infrequent. Bland foods add least to the constant trouble. Careful diet is practiced, and it does reduce to a minimum the distress.

Therefore, nutrition fails early; wasting follows rapidly and surely, though often more rapidly than lack of food would indicate (cancer poisoning). This rapid loss brings a dry skin, a pinched and wrinkled appearance. Paleness follows the emaciation, and blood loss completes the picture, which is cancer.

Vomiting is usually a prominent symptom and increases as the disease progresses. As compared to ulcer it is more irregular and more abundant, more frequently rancid, foul and obnoxious, and there is a longer interval between attacks of vomiting. Nausea and vomiting may be present when no food is in the stomach, and both conditions are easily excited by food and drink. Blood, bright or coffee grounds in character, is frequently found in the vomitus. There is less effort in cancer vomiting, and though the patient feels wonderful relief, it is not always complete. The characteristic of cancer vomiting is its irregular delayed type, with poorly macerated, undigested masses of food and large quantities of foul vomitus frequently containing blood. All this may be present and the outlet may not be badly stenosed (motor power is poor as a result of cancer infiltration). Eructations of liquids of a low acid content day or night are frequent means of some relief.

Gas is usually annoying, is increased over that in ulcer, is quite continuous, though there are irregular periods of intense belching generally soon after food. Especially is this true late, when diet is not strictly followed. More frequently in cancer than in ulcer the gas has a foul offensive odor, and the breath is bad, in contrast to the bland or sour breath of gastric ulcer.

As in ulcer, the bowels are constipated, but blood is more often present in the stools. Tumor is found in from sixty to seventy per cent of patients with cancer as they present themselves for relief, while in ulcer not more than five per cent show tumor. The test meal has its pointings: Lessened hydrochloric acid, increased blood, food remnants more constant, lactic and fatty acids oftener present. Therefore, the test meal should be considered, but it should be interpreted in the light of a careful clinical history.

SUMMARY.

In gallstones the general health does not suffer until complications are present. The course of ulcer is prolonged and fluctuating, periods of trouble and periods of perfect or partial health; :but in cancer it is short and steadily downward.

Pain in gallstones is irregular in time, of sudden onset, and of severe, short duration, abrupt cessation, radiates to right arch and back and is independent of food. In ulcer, pain is usually clear-cut, in spells, regular in time, and eased by food to again reappear in from two to four hours. In cancer the pain is continuous, dull, depressing and not only is not controlled by food, etc., but is immediately increased by it.

In gallstones the vomiting is less a factor in diagnosis. It is small in amount unless the attack is soon after a meal. It is usually bitter, sour bile and is not so often a means of relief, nor does it play any part in the state of nutrition (except during late complications). In ulcer, vomiting is as regular as is pain, and consists of sour material, not offensive and, if abundant, is often excessive in liquids. It brings complete relief and is controlled as is the pain. Vomiting in cancer is irregular, and the vomitus large in quantity of poorly macerated food mass. It is foul, often blood-stained and brings great though rarely complete ease.

In gallstones gas is troublesome only at the time of the colic; it may be excessive and give the distressing upward pressure, but immediately disappears on cessation of cramps. Perhaps this sensation is due less to gas than to the radiation of pain. In ulcer gas is a symptom at a time when other symptoms are present, and is controlled in the same manner that the other symptoms are controlled. In cancer the gas is continuous and increased in amount, with periods of great increase usually soon following food.

Blood is rare in gallstones, in ulcer it is quite rare (in about one-fourth of the cases), but in cancer it is so common (two-thirds of the cases) both by stomach test and fecal examination.

In gallstones the patient is normal physically, save when there are late complications with pancreatic disturbance. In ulcer the patient is hopeful and active, though often reduced in nutrition. In cancer the patient is depressed, languid, weakened, tired, melancholy, discouraged, pale and perhaps cachectic.

The diagnosis of gallstones is met with considerable satisfaction. That of peptic ulcer is perhaps less clearly defined but is rapidly approaching a degree of certainty. Both in gallstones and in ulcer, ignorance, neglect or willful delay find some excuse because the consequences are not so plainly demonstrable, and delay is not so often fatal. The diagnosis of cancer of the stomach is extremely difficult to make in that early stage when surgery, the only means of relief, offers a hope of cure, and when delay is fatal. Ignorance on the part of the physician is unpardonable; neglect, almost criminal. The physician's position is harassing; the patient's, perilous. Though late in his diagnosis, either because of insuperable circumstances, lack of knowledge or unpardonable neglect, the internist has met his responsibility, at least in a small measure, when he places his patient with gallstones, ulcer, or suspected gastric cancer in the hands of a competent surgeon, but the surgeon's responsibility does not cease with exploration or gastro-enterostomy alone, because careful resection is necessary when we are in the presence of cancer or any suspicious ulcerous lesion.

---

### THE PREVALENCE OF CANCER.

By Samuel G. Dixon, M. D., LL. D., Commissioner of Health of the State of Pennsylvania.

As a study of any subject is obviously more or less impossible from statistical data orally presented, I will take the liberty of merely calling your attention at this moment to some of the more general and most important facts concerning the prevalence of cancer, and leave the details of the statistical tables to your more leisurely consideration. From a purely scientific view point, the term cancer may be considered as somewhat indefinite and unsatisfactory, including as it does a considerable variety of neoplasms worthy of individual study, but which are too frequently grouped under the general term.

Statistically, deaths from cancer include those definitely returned as such, also those in which the nature of the new growth is either not definitely diagnosed or not definitely stated by the attending physician who may simply employ the term "malignant growth," or some such equivalent, upon the death certificate.

Therefore, so far as statistics are concerned, the term cancer may be considered as including all tumors which are not definitely stated to be of malignant nature. It is unfortunate that even this liberty must be taken with the individual returns of causes of death, but experience has proved that in order not to vitiate our statistics such inclusions must be made.

In view of the fact that some of the most powerful energies of medical science are at the present time being exerted in investigating the nature, origin and incidence of cancer, the importance of accurate and definite statistics upon the subject becomes of absolute necessity. In the Bureau of Vital Statistics in the Pennsylvania State Department of Health, it is necessary to ask each month not less than twenty-five physicians throughout the state for a more definite statement, even as to the location of cancers and malignant growths. It may not be inappropriate to remark here that while the information is in the majority of instances cheerfully supplied, in some others the inquiry is characterized as unnecessary or highly impertinent.

As the value of all mortality statistics must depend upon the honesty and accuracy of physicians who supply the information upon which these statistics are based, I know of no better time or opportunity to impress upon the members of this society the fact that they individually can very materially aid in the study of cancer by stating definitely upon all death certificates not only the organ or part affected, but whenever possible the nature of the neoplasm, such as carcinoma, sarcoma, epithelioma, etc.

A glance at the available statistics concerning cancer shows most conclusively that the mortality from this disease is steadily increasing throughout the civilized world.

In the registration area comprising about fifty per cent of the entire population of the United States, there were 30,514

deaths from cancer in all forms in 1907. We are, therefore, probably quite safe in assuming that over 50,000 deaths occurred from this disease in the entire United States during that year.

The death rate per 100,000 of population increased from 47.9 in 1890 to 73.1 in 1907. In England and Wales during the same period the rate increased from 63 to 91, in Ireland, from 43 to 76; in Scotland, from 62 to 94; in Prussia, from 41 to 73; in Italy, from 43 to 61; in Spain, from 39 to 47; in Hungary, from 27 to 42; in the Netherlands, from 70 to 102, and in Switzerland, from 114 to 132. The statistics for Pennsylvania show that the rate in 1890 was 41.5, and in 1907 it was 62.8, in neither instance being quite so high as the general rate for the entire country, but nevertheless sharing consistently in the steady and average increase.

If the proper correction is made for the distribution of population by age periods, a most important factor in determining comparative death rates from this disease, the average for the several states in this country, which apparently present some rather wide variations, would be found to possess considerable uniformity.

The distribution between urban and rural localities shows no marked variations, the apparently high rates for some of our larger municipalities being accounted for by the fact that many cases of cancer from the hospitals in cities and the deaths occurring therein are so registered.

The International Classification of causes of deaths makes seven subdivisions of cancer, according to locality or organ affected. These groups with the percentages of deaths to total deaths from cancer are as follows:

1. Cancer of the mouth . . . . . . . . . . . . . . . . . . . . . . . . . . 3.2
2. Cancer of the stomach and liver . . . . . . . . . . . . . . . . . 38.0
3. Cancer of the intestines and peritoneum . . . . . . . . . . 11.7
4. Cancer of the female genital organs . . . . . . . . . . . . . . 14.3
5. Cancer of the breast . . . . . . . . . . . . . . . . . . . . . . . . . . 8.5
6. Cancer of the skin . . . . . . . . . . . . . . . . . . . . . . . . . . . 3.7
7. Cancer of the other organs or organs unspecified. . . . 20.6

SEX

In the aggregate, deaths from cancer are more frequent in females. If, however, we exclude cancers of the mammary and generative organs and base our comparison upon the organs or localities of the body which may be equally affected without regard to sex, we find the deaths of males largely in excess.

The mammary and generative organs are affected in about forty per cent of the deaths among females, and about an equal percentage is due to cancer of the stomach, liver and intestines combined, while among males the latter organs are affected in at least fifty-three per cent of all cases.

AGE.

While ninety-six per cent of all deaths from cancer occur in persons over thirty-five years of age, it is interesting to note that the death rate for children under five years of age is greater than the rates between the years of five and fourteen.

Among 207,764 deaths from cancer compiled by the registrar general's office of England, 749 occurred in children under five years of age. Of this number 240, or thirty-two per cent, were due to cancer of the kidney and suprarenal glands, and 104, or fourteen per cent, were due to cancer of the eye or orbit; proportions which are far in excess of the rates at any subsequent quinquennial age period for those organs or localities.

COLOR.

Cancer does not seem to prevail to the same extent among the negroes of this country as it does among the whites. Vital statistics are exceedingly meager in many portions of the South where the negroes form a very large proportion of the population

In those localities, however, in which registration is quite accurate, the difference appears well marked. In Washington, D. C., the rate per 100,000 of each color is white, 102.0; black, 63.4. In Louisville, Ky., the white rate is 54.9; black, 29.6. In New Orleans, La., the white rate is 87.1; black, 77.2. In Baltimore, Md., the white rate is 89.4; black, 68.3. In Memphis, Tenn., the white rate is 59.1; black, 33.4, the average difference for the five places just mentioned being 22.1 per 100,000 of population.

CONJUGAL CONDITION.

A comparison between the death rates of the single, married and widowed at the various ages shows that the rate for the widowed of both sexes is higher at all ages than either the single or married, and that the rate for the single of both sexes is higher than the married at all ages over forty-five years.

NATIVITY.

The death rates in the United States according to the nativity of mothers form quite an interesting comparison with the death rates of the foreign countries represented. Italy, Hungary and Russia present the lowest death rates in Europe, and the immediate descendants of mothers from these countries present the lowest death rates in the United States, and very considerably lower than the general death rate for the entire country.

A reference to the statistical tables will show the proportion of deaths from cancer for certain localities by sex and age per 1000 deaths from all causes at corresponding ages, and represents a more detailed subdivision than the group of the International Classification before mentioned.

From this table it will be noticed that cancer of the abdomen, excluding cancer of the stomach, liver, bladder, uterus and ovaries, was more frequent in males than in females and most prevalent between the ages of twenty and forty-four years. Cancer of the bladder was more frequent in males than females at all ages with the greatest prevalence over sixty-five years of age. Cancer of the brain was more frequent in males at all ages and greatest at the age period of twenty to forty-four years. Cancer of the eye was more frequent in males at all ages and greatest at the age periods over sixty-five years. Cancer of the ear, face and neck was more frequent in males at all ages and greatest at the age periods over sixty-five years. Cancer of the larynx was more frequent among males at all ages and greatest at the age period of forty-five to sixty-five years. Cancer of the liver was more frequent in males at all ages under sixty-five years. Over that age it was more prevalent in females, the highest rate being for males at the age period of twenty to forty-four years. Cancer of the lungs was more frequent in males at all ages and greatest at the age period of forty-five to sixty-four years. Cancer of the mouth was more frequent in males at all ages and greatest over sixty-five years of age. Cancer of the rectum was more frequent in males at all ages and greatest at the age period of twenty to forty-four years. Cancer of the stomach was more frequent in males at all ages and greatest at the age period of forty-five to sixty-four years. Cancer of the uterus was more prevalent at the female age periods of twenty to forty-four years; and of the breast, at ages over sixty-five.

The statistics of occupation in relation to cancer do not appear to associate an excessive proportion with any individual occupation.

These few facts, meager as they are in connection with the tabular data of this very important cause of death, should be sufficient to demonstrate the fact that if statistics are to furnish any clue to the cause of cancer, or are to aid in any way in its prevention, there must be an unselfish and painstaking effort on the part of physicians to supply full and definite information concerning each individual case. This is imperative in order that proper weight may be given to every factor in final analysis, as it may be in the correlation of identical facts concerning a large number of deaths that we may at least find a guide to fruitful research.

CONJUGAL CONDITION.

Death rates per 100,000 of population.

| | 15 to 44 yrs. | | | 45 to 64 yrs. | | | Over 65 yrs. | | |
|---|---|---|---|---|---|---|---|---|---|
| | S. | M. | Wd. | S. | M. | Wd. | S. | M. | Wd. |
| Total ----- | 8.7 | 28.6 | 69.6 | 192.1 | 175.6 | 256.4 | 518.7 | 426.0 | 455.8 |
| Males ---- | 6.5 | 15.7 | 40.3 | 142.1 | 125.8 | 200.8 | 512.5 | 388.0 | 433.7 |
| Females -- | 11.3 | 39.8 | 80.7 | 248.8 | 238.8 | 275.9 | 523.1 | 496.7 | 464.9 |

### CANCER IN THE UNITED STATES.

Rates per 100,000 of population.

| | 1900 | 1901 | 1902 | 1903 | 1904 | 1905 | 1906 | 1907 |
|---|---|---|---|---|---|---|---|---|
| Registration area | 63.0 | 64.5 | 65.5 | 68.6 | 70.6 | 72.1 | 70.8 | 73.1 |
| Connecticutt | 68.7 | 70.3 | 68.3 | 76.4 | 68.8 | 75.9 | 80.6 | 80.1 |
| Indiana | 42.8 | 44.1 | 47.9 | 49.3 | 50.4 | 55.3 | 53.7 | 57.1 |
| Maine | 74.6 | 82.4 | 86.7 | 85.0 | 86.3 | 92.9 | 86.2 | 101.3 |
| Massachusetts | 74.6 | 76.3 | 76.5 | 80.9 | 88.0 | 89.3 | 90.3 | 93.5 |
| Michigan | 61.2 | 60.0 | 59.6 | 67.5 | 67.4 | 64.2 | 67.6 | 66.7 |
| New Hampshire | 71.9 | 87.7 | 81.2 | 77.5 | 80.4 | 83.7 | 89.2 | 95.8 |
| New Jersey | 53.9 | 58.4 | 54.0 | 58.3 | 57.6 | 63.2 | 66.1 | 65.4 |
| New York | 66.7 | 70.1 | 69.5 | 71.7 | 73.8 | 76.1 | 76.2 | 78.9 |
| Pennsylvania | 41.5 | | | | | | 60.7 | 62.8 |
| Rhode Island | 70.5 | 73.0 | 83.1 | 77.3 | 86.6 | 80.4 | 78.3 | 91.1 |
| Vermont | 87.9 | 70.5 | 69.1 | 93.7 | 87.0 | 84.2 | 85.3 | 99.0 |

Between the years 1900 and 1906, the rates for the principal foreign countries were as follows, per 100,000 population:—

| | 1900 | 1901 | 1902 | 1903 | 1904 | 1905 | 1906 |
|---|---|---|---|---|---|---|---|
| England and Wales | 82.9 | 84.2 | 84.4 | 87.2 | 87.9 | 88.5 | 91.7 |
| Scotland | 80.0 | 82.0 | 83.0 | 84.0 | 84.7 | 88.4 | |
| Ireland | 60.0 | 65.0 | 65.0 | 69.1 | 69.4 | 74.9 | 79.3 |
| Germany | 72.0 | 75.0 | 75.0 | 77.4 | 80.0 | 80.9 | |
| Norway | 91.0 | 95.0 | 88.0 | 93.2 | 96.0 | 98.4 | |
| Hungary | 37.0 | 36.0 | 38.0 | 39.1 | 40.6 | 40.2 | 40.4 |
| The Netherlands | 92.0 | 94.0 | 95.0 | 99.0 | 97.9 | 101.2 | 100.7 |
| Switzerland | 130.0 | 128.0 | 127.0 | 131.0 | 130.3 | 131.8 | |
| Spain | 39.0 | 42.0 | 43.0 | 44.2 | 46.8 | 46.8 | 48.0 |
| Italy | 52.0 | 53.0 | 54.0 | 54.0 | 56.9 | 58.0 | 61.3 |

### PROPORTIONS OF DEATHS FROM CANCER OF CERTAIN ORGANS PER 1,000 DEATHS FROM ALL CAUSES AT CERTAIN AGES.

| Locality | All Ages | | | 20 to 44 Years | | | 45 to 64 Years | | | 65 years and over | | |
|---|---|---|---|---|---|---|---|---|---|---|---|---|
| | Total | M. | F. | Total | M. | F. | Total | M. | F. | Total | M. | F. |
| Abdomen | 82.8 | 92.4 | 76.9 | 78.6 | 118.9 | 62.6 | 79.2 | 92.2 | 71.5 | 88.4 | 83.3 | 92.4 |
| Bladder | 13.5 | 25.3 | 6.2 | 5.8 | 6.3 | 5.6 | 12.3 | 22.8 | 6.0 | 19.3 | 34.7 | 6.9 |
| Brain | 2.0 | 2.6 | 1.7 | 4.4 | 4.7 | 4.3 | 1.1 | 1.6 | 0.7 | 1.4 | 2.0 | 0.8 |
| Breast | | 7.5 | 157.8 | | 11.0 | 151.9 | | 7.8 | 157.8 | | 6.1 | 162.4 |
| Eye | 1.3 | 2.4 | 0.7 | | | | 0.3 | 0.4 | 0.2 | 2.5 | 4.6 | 0.8 |
| Genitals | | 9.6 | 6.3 | | 3.1 | 8.4 | | 8.6 | 5.8 | | 12.8 | 6.1 |
| Head, face and neck | 59.2 | 104.2 | 31.4 | 37.3 | 92.3 | 15.5 | 46.2 | 95.1 | 17.4 | 87.9 | 118.0 | 63.9 |
| Larynx | 4.9 | 10.8 | 1.3 | 4.0 | 7.8 | 2.5 | 5.4 | 13.1 | 1.0 | 4.8 | 9.2 | 1.2 |
| Liver | 133.4 | 145.6 | 125.9 | 112.3 | 159.6 | 93.6 | 133.7 | 151.4 | 123.3 | 143.2 | 132.4 | 151.9 |
| Lower extremities | 2.7 | 3.7 | 2.1 | 2.7 | 4.7 | 1.9 | 2.0 | 2.4 | 1.7 | 3.6 | 4.6 | 2.9 |
| Lungs | 5.5 | 6.1 | 5.1 | 4.9 | 6.3 | 4.3 | 7.4 | 8.6 | 6.7 | 2.9 | 3.1 | 2.9 |
| Mouth, tongue and throat | 46.8 | 95.5 | 16.8 | 25.3 | 67.3 | 8.7 | 45.4 | 98.3 | 14.2 | 59.6 | 101.2 | 26.5 |
| Ovaries | | | 9.7 | | | 16.7 | | | 8.9 | | | 5.3 |
| Rectum | 42.9 | 54.9 | 35.5 | 54.2 | 79.8 | 44.0 | 39.8 | 50.6 | 33.5 | 42.6 | 53.7 | 33.8 |
| Stomach | 315.6 | 430.6 | 244.7 | 249.6 | 427.2 | 179.2 | 318.8 | 441.4 | 246.4 | 348.0 | 422.6 | 288.7 |
| Upper extremities | 1.9 | 3.3 | 1.1 | 1.3 | 1.6 | 1.2 | 0.9 | 1.2 | 0.7 | 3.9 | 0.6 | 1.6 |
| Uterus | | | 276.8 | | | 399.9 | | | 304.2 | | | 151.9 |

Page(s) missing

Page numbers 50-51 omitted text is complete

NATIVITY.

A comparison of the death rates according to birth place of mothers shows the following, per 100,000 of white population:—

| | |
|---|---|
| United States | 48.3 |
| Ireland | 76.4 |
| Germany | 78.2 |
| England and Wales | 72.0 |
| Scandinavia | 31.1 |
| Scotland | 81.8 |
| Italy | 22.8 |
| France | 92.8 |
| Hungary | 31.5 |
| Russia | 25.7 |

AGE.

Death rate by sex at certain ages per 100,000 of population at corresponding ages:

| | Under 5 | 5 to 14 | 15 to 44 | 45 to 64 | Over 65 |
|---|---|---|---|---|---|
| Total | 1.3 | 0.8 | 20.5 | 194.8 | 454.3 |
| Males | 1.5 | 0.7 | 11.5 | 137.6 | 417.0 |
| Females | 1.1 | 0.9 | 29.4 | 253.0 | 487.6 |

REFERENCES.

United States Census Office Reports.
Registrar General's Office Report, England.
Report of Imperial Cancer Research Fund.

---

### BREAST AND BEST OPERATIVE TECHNIC.

By William L. Rodman, M. D., Philadelphia.

In diagnosing a tumor of the breast the three most important considerations are the age of the patient, the location of the growth, and its adherence or nonadherence to the surrounding parts.

AGE.

While carcinoma is certainly more common in women over forty years of age, there are enough cases in younger women to discredit any diagnosis made on this basis alone. I made a careful analysis of five thousand cases and found that over one-fifth of them occurred in women under forty.

Although S. W. Gross reported but one case twenty-eight years of age, I have operated on six cases between twenty-three and twenty-eight; Park and Warren have each reported cases aged twenty-two; Richardson, one aged twenty-one; and A. J. McCosh, one aged nineteen.

The period of greatest liability to cancer of the breast is about and after the climacteric, when the epithelial elements of the gland are apt to proliferate unduly and functional decline is taking place. Involution mastitis, which is so difficult to distinguish from carcinoma, is also apt to occur about the menopause. This should always be kept in mind. An inflammatory process is suggested by the irregular and variable swelling and probable painfulness caused by mastitis.

SITE OF GROWTH.

Cancer, though affecting all parts of the breast, is more often found in its auxiliary than in its sternal half; and the upper outer quadrant is affected more frequently than the lower. That portion of the gland directly behind the areola is next in point of frequency to be involved. When thus situated, by its adhesions to surrounding structures it pulls upon and causes retraction of the nipple.

Sarcoma and benign tumors, on the other hand, are more frequently found in the sternal hemisphere, particularly in the upper and inner quadrant. These facts are, perhaps, of greater importance in diagnosis than the age of the patient.

MOVABLENESS.

The fact of greatest diagnostic importance in a tumor is the degree to which it is movable. A growth which is not adherent to the skin and is freely movable is almost certainly not cancerous. It may be malignant if disconnected from the skin and only slightly movable. If the tumor has caused either retraction of the nipple or dimpling of the skin and is at the same time immovable, it is almost unquestionably carcinomatous. Perhaps too much emphasis has been laid on retraction of the nipple as a means of diagnosis, as it occurs in but fifty-one per cent of all cases. The skin over the tumor is somewhat roughened and wrinkled, and if pinched between the thumb and forefinger will nearly always dimple. A more delicate test, however, is to expose both breasts fully and move each in every direction until an asymmetry over the tumor shows by contrast as the result of pulling on the skin if the trabeculæ are shortened to any extent whatever. This is a very valuable sign, and in small, deep-seated, or retromammary growths will be found a very expedient method.

There are many other points which have a certain value diagnostically, but they are too indefinite to influence one seriously. Married women are more frequently affected than unmarried, and women who have had children more often than those who have not.

I am of opinion that heredity can not even be inferred in more than twenty-five per cent of all mammary carcinomata; still its influence should not be ignored. I have only once operated on mother and daughter; the latter in June, 1904, and the former in July, 1906.

In about ten per cent of cases, cancer of the breast, in its early stage, can not be recognized clinically, for, added to the difficulty of distinguishing between solid growths, there is the greater one of differentiating benign and malignant cysts. No one will deny that it is most necessary to separate the one from the other, and it can only be done safely by the microscope.

I would emphasize the fact that cysts are often malignant, and that the degree of their malignancy is very great, when they would seem macroscopically, on account of their thin walls and clear contents, to be benign. While it is true that a sanguinolent discharge indicates malignancy, a clear fluid most certainly does not mean that the tumor is benign. The danger of any other treatment than excision of the entire cyst wall as well as enough of the surrounding tissue practically to insure extirpation of the disease, is great, as a mistake made at the operation probably will not be atoned for by a second and more complete one after the microscopic report. The patient will generally not allow a second operation, and even should she permit it, it will quite likely result in failure on account of epithelial cells having been liberated at the time of the first operation.

The safest way is to remove the most suspicious part of the growth, along with the adjacent structures, and give it at once to a competent microscopist, who is present, then cauterize the wound with the actual cautery and plug it with gauze. The danger of disturbing cancer cells can be obviated only in this way.

TREATMENT.

Operation is the only treatment for cancer of the breast. Local applications, radium, x-rays and caustics are futile and lessen the chances of a cure, which depend largely on the timeliness with which operation is performed.

It will be well to consider a few of the fundamental principles of the operation for mammary carcinoma. The earlier and more radical the procedure the better. Practically all progressive surgeons admit that a large wound, removal of the pectoral muscles, and a complete axillary dissection en masse are necessary. A few who do not remove the muscles cut across and retract the ends, so as to uncover the axilla, subsequently suturing them. A small number remove the fasciæ covering the subscapular, serratus, latissimus and other muscles exposed in the axillary dissection, and still fewer continue the incision far enough to expose the sheath of the rectus in the epigastric triangle. A very few remove it, though it should always be done.

Carcinoma spread peripherally by the penetration of its epithelial cells along the deep facial planes, consequently the reason for removing possibly infected fasciæ will at once be appreciated. There is much to give credence to the opinion which has been expressed, that thoracic metastases are less frequent than abdominal ones; that the liver is more often the site of secondary foci than the lungs, and that the sheath over the upper part of the rectus should be removed to prevent infection traveling from the breast to the liver. Since hearing these views expressed by Mr. Handley in 1904, I have enlarged internally the wound that I had been in the habit of making, doing a freer dissection of the epigastric triangle, and removing the sheath of the rectus in all cases where the sternal hemisphere, especially the lower quadrant, is involved. Growths in the outer half of the breast metastasize to the axilla, where it is possible to eradicate them, but the ones in the inner half are far more deadly, as they metastasize to the liver, lungs, mediastinum, sternum, ribs or vertabræ where surgical attack is impossible.

The advantages of first attacking the axilla are evident and not sufficiently appreciated; indeed, a large majority of surgeons still plan their incisions so as to work from sternum to axilla, in spite of the dangers incident to this method. The advantages of working from axilla to sternum are as follows: First, the axilla may be so deeply involved that any attempt at removal would be fruitless, and the sooner this is ascertained the better. Second, the blood vessels can be reached and tied at their origin, hence the same vessel is not cut several times, as is necessarily done in working from sternum to axilla; therefore, shock and hemorrhage are greatly lessened. Third, it is both easier and better to dissect the axillary space from above downward, as the dissection is begun beyond the boundaries of the disease. Fourth, the great danger of expressing cancer cells, as a result of manipulating the infected mamma and lymph nodes, to surrounding tissues, even to remote organs, is largely, if not wholly, avoided. Handling of the infected breast is dangerous. Fifth, if the work is begun at the sternum a dissection en masse is sometimes made impossible, as the mass will pull on and break the axillary tail if not held by an assistant; this should be avoided. Greater exactness is possible if the incision is begun at the axilla, and therefore use of the arm will be less interfered with. I consider it of the greatest importance not to allow the incision to extend onto the arm, but to confine it to the chest, as the cicatrix resulting from an incision carried onto the arm frequently hampers its movements. The axilla is reached by a straight clavicle to an inch or less below the tendon of the pectoralis major and two finger breadths internal to the sulcus between arm and chest. This permits perfect access to and work in the axilla and interferes in no way with the future usefulness of the arm. Early and free removal before metastases have occurred to neighboring structures should insure a permanent cure in eighty per cent of all cases. (To be continued.)

